# The spatial variation of O_3_, NO, NO_2_ and NO_*x*_ and the relation between them in two Swedish cities

**DOI:** 10.1007/s10661-017-5872-z

**Published:** 2017-03-13

**Authors:** Annika Hagenbjörk, E. Malmqvist, K. Mattisson, Nilsson J. Sommar, L. Modig

**Affiliations:** 1 Division of Occupational and Environmental Medicine, Department of Public Health and Clinical Medicine, Umeå University, SE-90187 Umeå, Sweden; 2 Division of Occupational and Environmental Medicine, Department of Laboratory Medicine, Lund University, SE-221 85 Lund, Sweden

**Keywords:** Ozone, Nitrogen oxides, Diffusive sampler, Measurements, Spatial variation, Determinants of exposure

## Abstract

Ozone and nitrogen oxides (NO_*x*_) are air pollutants with known associations to adverse health effects on humans. Few studies have simultaneously measured ozone and nitrogen oxides with high spatial resolution. The main aim of this paper was to assess the levels and variation of ground-level ozone, NO_2_ and NO_*x*_ in two Swedish cities. An additional aim was to describe the levels of these pollutants within and between three different types of measurement sites (regional background, urban background and traffic sites) and within and between different measurement periods of the year. Three weekly sampling campaigns of NO_*x*_ and ozone were conducted simultaneously at 20 sites in two Swedish regions using Ogawa badges. Ozone was measured at 20 additional sites in each area. The median ozone concentration for all measurements was statistically significantly higher in Malmö (67 μg/m^3^) compared to Umeå (56 μg/m^3^), and in both cities, ozone levels were highest in April. Measurement period was a more important factor for describing the variation in ozone concentrations than the type of measurement site. The levels of NO_2_ and NO_*x*_ were statistically significantly higher in the Malmö area (8.1 and 12 μg/m^3^) compared to the Umeå area (4.5 and 8.9 μg/m^3^). The levels were generally highest at the sites categorized as traffic, while the variability between different seasons was sparse.

**Electronic supplementary material:**

The online version of this article (doi:10.1007/s10661-017-5872-z) contains supplementary material, which is available to authorized users.

## Introduction

Ozone (O_3_) and nitrogen oxides (NO_*x*_) comprising nitrogen dioxide (NO_2_) and nitric oxide (NO) are among the most important contaminants in urban areas, as they have been associated with adverse effects on human health and the natural environment.

Ozone is an important oxidant in the troposphere and is formed through chemical reactions in the presence of NO_*x*_ and volatile organic compounds (VOCs), under the influence of solar radiation. The reactions between NO, NO_2_ and O_3_ in the atmosphere is theoretically a “null cycle” with no net production or destruction of O_3_, as the effect of reaction 2 is the reverse of reaction 1:

1 $$ \mathrm{NO}+{\mathrm{O}}_3\to {\mathrm{NO}}_2+{\mathrm{O}}_2 $$

2 $$ {\mathrm{NO}}_2+\mathrm{hv}+{\mathrm{O}}_2\to \mathrm{NO}+{\mathrm{O}}_3 $$

To generate a net production of ozone, NO has to be converted to NO_2_ without consuming ozone. In the presence of VOCs in the atmosphere, through reactions with NO and atmospheric peroxides (RO_2_), this can be accomplished, leading to an accumulation of ozone. The chemical coupling between O_3_ and NO_*x*_ through reactions 1 and 2 results is an indissoluble link between NO_2_ levels and O_3_ levels, which implicates a reduction in O_3_ concentrations at high NO concentrations. Ozone production is dependent on the state of NO_*x*_, as NO_2_ and NO increase the production and dissociation of O_3_, respectively. Consequently, an increased NO/NO_2_ ratio reduces the ozone concentration (Melkonyan and Kuttler 2012).

The levels of ozone at a specific location is in brief dependent on the concentration of ozone in the free troposphere, long-range transport of ozone and its precursor emissions and locally produced ozone. Important sinks of ozone are local depletion by reactions with NO in the vicinity of NO_*x*_ emissions (i.e. urban areas) and deposition of ozone to the ground (WHO 2008). Biogenic volatile organic compounds (BVOCs ), e.g. isoprenes and terpenes, emitted from plants, also play an important role in the formation of secondary pollutants such as ozone (Oderbolz et al. 2013). The background level of ozone, defined as the ozone concentration in a given area that is not assignable to local anthropogenic sources, has an annual variation and differs with latitude and altitude (Vingarzan 2004). On a global scale, current levels of background ozone have increased approximately two times compared to the levels measured over a century ago (Vingarzan 2004). This is explained by influence from human activities on ozone levels in parallel with industrial development. Over the past three decades, the increasing ozone trend has declined or remained constant, probably due to decreasing NO_*x*_ emissions in Europe and North America (Vingarzan 2004). Background sites in the Northern Hemisphere have shown a spring maximum peaking in May with an increasing concentration in a south to north direction at the latitudinal range 10–60° (Vingarzan 2004). Ozone is known to have a large-scale spatial variation, but studies in Sweden and Great Britain have shown that there is also a considerable local spatial variability driven by local emissions and meteorology (Coyle et al. 2002; Klingberg et al. 2012; Sundberg et al. 2006). Coastal sites, e.g. within a few kilometres from the coast, have shown higher ozone levels than inland sites due to low deposition velocity of ozone over water (Entwistle et al. 1997; Klingberg et al. 2012; Piikki et al. 2009).

The major source of NO_*x*_ in urban areas is motor vehicle exhaust. The main proportion of the NO_*x*_ is emitted as NO, while a smaller proportion is emitted directly as NO_2_. Even though the total amount of NO_*x*_ has a downward trend in Europe, the NO_2_ share of NO_*x*_ emissions has increased in recent years and is dependent on the vehicle, fuel type, exhaust treatment technology and driving conditions (Carslaw 2005).

Ozone and NO_2_ levels have been studied for many locations in the world (Clapp and Jenkin 2001; Garcia et al. 2005; Im et al. 2013; Mazzeo et al. 2005; Melkonyan and Kuttler 2012; Notario et al. 2013; Song et al. 2011; Syri et al. 2001). Often, the measurements are performed by means of continuous on-line monitors at one measurement site. However, these monitors cannot provide information on concentrations at a finer scale, to provide the spatial variations in levels over a greater geographical area. Diffusive samplers are an ideal tool to study the spatial distribution of the concentration of certain compounds as they are inexpensive and require no power. Diffusive samplers are, unlike on-line monitors, easily deployed at many sites simultaneously and give the opportunity to carry out surveys over wide geographical areas to obtain the spatial distribution.

This work is part of a study designed to develop a Land Use Regression (LUR) model for ozone exposure to be used in epidemiological studies (Malmqvist et al. 2014). In doing this, the local spatial variation of ozone in two study areas, Malmö and Umeå, in different parts of Sweden was evaluated. The Land Use Regression study also included simultaneous measurements of NO_2_ and NO_*x*_.

The main aim of this paper was to assess the levels and variations in levels of ground-level ozone, NO_2_ and NO_*x*_ between and within two Swedish cities, during three different measurement periods and at three different types of measurement sites (*regional background sites*, *urban background sites* and *traffic sites*). Another objective was to estimate how type of measurement site and measurement period influenced the variability in concentrations and the ratio between selected pollutants. To our knowledge, there is no study that has measured ozone and NO_*x*_ simultaneously at so many sites (*n* = 20), with repeated measurements in three periods (April, May/June and August) in two parts of a country.

## Materials and methods

### Study areas

**Fig. 1:**
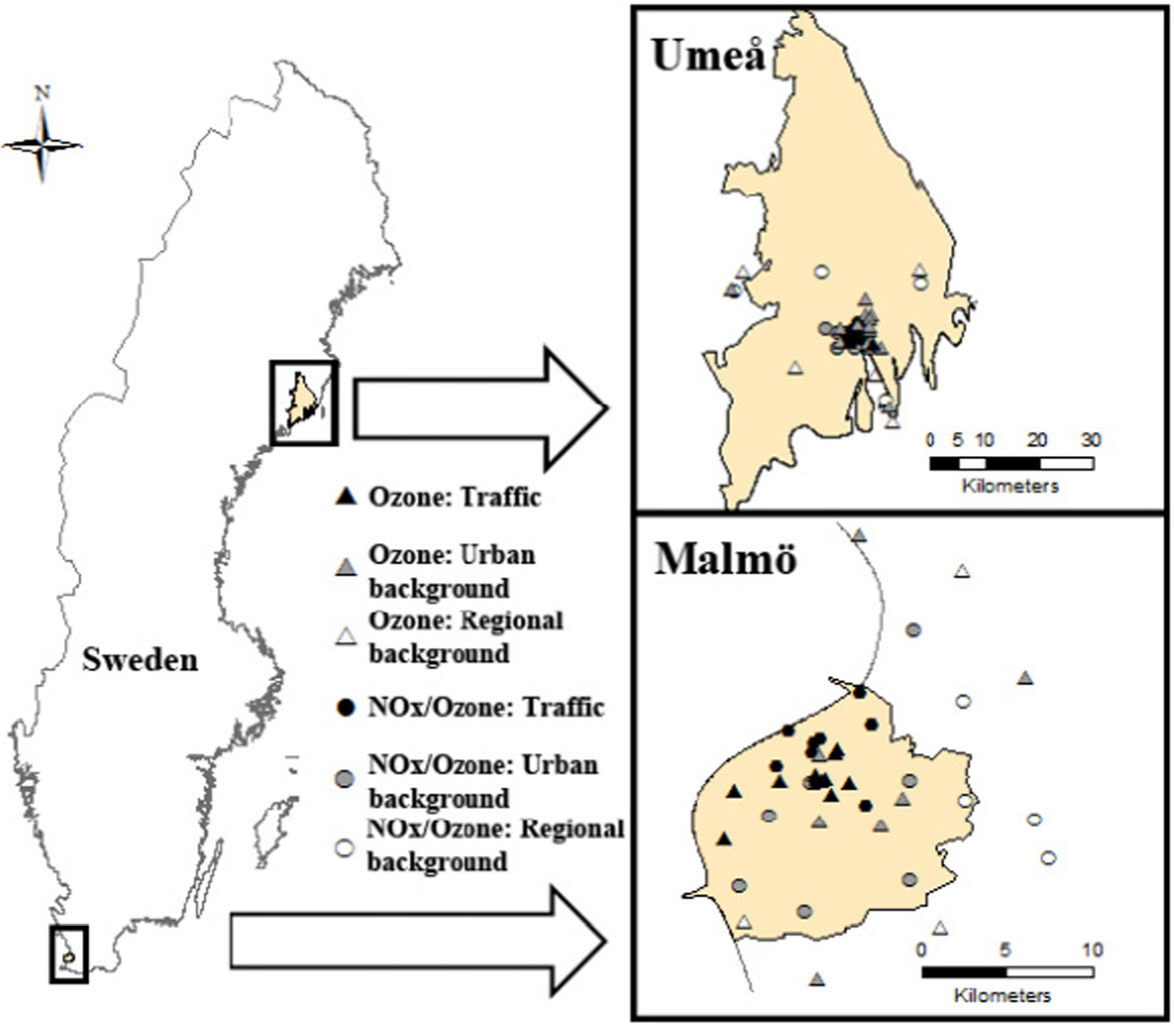
The two study areas showing the location of the sampling measurement sites in each area. *Triangular dots* show the location of the 40 ozone measurement sites in each area. *Circular dots* show the location of the 20 NO_*x*_ + ozone measurement sites in each area. *Black dots* represent traffic sites; *grey dots* represent urban background sites; *unfilled dots* represent regional background sites

The sampling campaigns were conducted simultaneously in two Swedish regions approximately 1250 km geographically apart from each other (Fig. 1); Malmö, on the south-west coast (55° 36′ N, 13° 00′ E, elevation 18 m), with about 330,000 inhabitants, and Umeå with approximately 120,000 inhabitants, on the northeast coast (63° 50′ N, 20° 15′ E, elevation 14 m). The climate between the regions differs, with a milder climate in the Malmö region (mean annual temperature: 9.0 °C) and a colder climate in the Umeå region (mean annual temperature: 3.8 °C) (SMHI, Swedish Meteorological and Hydrological Institute 2016).

### Sampling equipment and methodology

Ogawa diffusive samplers (Ogawa & Company, Pompano Beach, FL, USA) were used to monitor ozone, NO_2_ and NO_*x*_. The sampling and the subsequent ion chromatography analysis of NO_2_, NO_*x*_ and ozone, respectively, have been described in detail previously (Hagenbjork-Gustafsson et al. 2010; Malmqvist et al. 2014). In short, the Ogawa sampler has a cylindrical body with two ends, which enables simultaneous monitoring of NO_2_ and NO_*x*_. Each end holds a collection filter, coated with a reactive chemical, one for trapping NO_2_ and the other for trapping NO_*x*_. NO is calculated as the difference between the NO_*x*_ and the NO_2_ concentration. For ozone measurements, another Ogawa sampler, provided with a collection filter for ozone, was used and deployed in the direct vicinity of the NO_2_/NO_*x*_ sampler. All measurements were performed by diffusive samplers as weekly averages of NO_2_, NO_*x*_ and ozone, respectively, and co-located measurements of these substances were taken at 20 sites in each study area. Ozone measurements were taken at 20 additional sites in each study area. The measurement sites were categorized in three groups: regional background sites, urban background sites and traffic sites depending on traffic density, distance from major roads, distance from other roads and population density, according to the criteria by EUROAIRNET. A more detailed description of the categorization and the study area has been given elsewhere (Malmqvist et al. 2014).

Three sampling campaigns were carried out in 2012; 16–24 April; 28 May–4 June; 20–27 August. The Ogawa samplers were mounted at about 2.5 m above ground level. All samplers were prepared and analysed at the division of Occupational and Environmental Medicine, Umeå University, Umeå. The coated filters were supplied by the manufacturer (Ogawa, USA).

### Statistical analysis

Differences in concentrations of ozone, NO, NO_2_ and NO_*x*_ between cities, type of measurement site and measurement period were tested using the Kruskal-Wallis test and Mann-Whitney *U* test. Linear regression was used to assess the relation between these air pollutants and to determine the amount of the total variance explained by the determinants city, type of measurement site and measurement period. The analyses were stratified by city, and differences in intercept and slope were tested by incorporation of an interaction term. Nitric oxide concentrations were log transformed in the analyses of determinants. A *p* value of less than 0.05 was considered to be statistically significant. Statistical analyses were carried out using R version 2.14.0 (R Core Team 2017).

## Results

The mean temperatures and wind speed during the three measurement periods were in Malmö in April 7.4 °C and 3.6 m/s, in May/June 11.8 °C and 3.6 m/s and in August 18.2 °C and 2.8 m/s, respectively, and in Umeå in April 4.0 °C and 2.8 m/s, in May/June 9.0 °C and 4.0 m/s and in August 12.4 °C and 3.3 m/s (wind data missing), respectively (SMHI 2016). During the third measurement period, 96 h of wind data for Umeå were missing between 23rd and 27th of August.

During the three measurement periods, Malmö had prevailing winds from SSE in April, from SW in May/June and from SSW in August. Umeå had predominant winds from SE in two of the measurement periods (April and August) and from S in the May/June measurement period (SMHI 2016).

### Ozone

The median ozone concentration for all sites and all measurements (*n* = 120 in each city) was statistically significantly higher in Malmö (67 μg/m^3^) compared to Umeå (56 μg/m^3^) (*p* < 0.001) (Table 1, Supplementary Table S1, Supplementary Table S2).

**Table 1:** Arithmetic mean (AM), standard deviation (SD) and median (min–max) concentrations of O_3_, NO_2_, NO_*x*_ and NO in different environments for all measurements in Malmö and Umeå

		Malmö				Umeå			
		Regional background sites (*n* = 21)	Urban background sites (*n* = 42)	Traffic sites (*n* = 57)	All measurements (*n* = 120)	Regional background sites (*n* = 27)	Urban background sites (*n* = 57)	Traffic sites (*n* = 36)	All measurements (*n* = 120)
O_3_ (μg/m^3^)	AM (SD)	72.5 (10.5)	68.5 (7.7)	65.8 (6.8)	67.9 (8.1)	54.3 (16.4)	52.3 (14.3)	49.3 (13.5)	51.8 (14.6)
	Median (min–max)	73.9 (51.7–95.2)	68.6 (51.9–84.5)	66.4 (51.2–84.2)	67.1 (51.2–95.2)	57.2 (27.5–93.3)	56.6 (29.4–71.8)	54.4 (26.5–69.7)	56.1 (26.5–93.3)
		Regional background sites (*n* = 12)	Urban background sites (*n* = 21)	Traffic sites (*n* = 27)	All measurements (*n* = 60)	Regional background sites (*n* = 13)	Urban background sites (*n* = 17)	Traffic sites (*n* = 30)	All measurements (*n* = 60)
NO_2_ (μg/m^3^)	AM (SD)	5.9 (2.8)	6.8 (2.5)	11.9 (4.3)	8.8 (4.3)	2.0 (1.8)	4.8 (2.1)	7.9 (7.9)	5.7 (6.2)
	Median (min–max)	5.2 (2.8–11.4)	6.6 (2.8–13.2)	11.2 (4.8–21.5)	8.1 (2.8–21.5)	1.6 (0.5–7.7)	4.4 (1.7–9.5)	6.1 (2.9–40.4)	4.5 (0.5–40.4)
NO_*x*_ (μg/m^3^)	AM (SD)	9.0 (2.2)	10.6 (2.6)	19.3 (6.8)	14.0 (6.7)	5.4 (1.2)	9.7 (4.6)	19.3 (23.0)	13.5 (17.4)
	Median (min–max)	9.2 (4.0–12.0)	9.5 (7.1–16.7)	17.6 (11.4–37.5)	12.0 (4.0–37.5)	5.4 (3.2–8.2)	8.5 (4.2–23.5)	12.2 (6.2–103.6)	8.9 (3.2–103.6)
NO (μg/m^3^)	AM (SD)	3.3 (2.1)	4.0 (2.5)	7.4 (4.3)	5.3 (3.8)	3.7 (1.3)	4.9 (2.9)	11.3(15.4)	7.9 (11.5)
	Median (min–max)	3.9 (0.6–5.6)	4.2 (0.6–11.2)	7.3 (0.6–18.6)	5.0 (0.6–18.6)	3.8 (0.6–5.4)	4.3 (0.6–14.0)	6.3 (1.8–63.2)	4.9 (0.6–63.2)

**Fig. 2:**
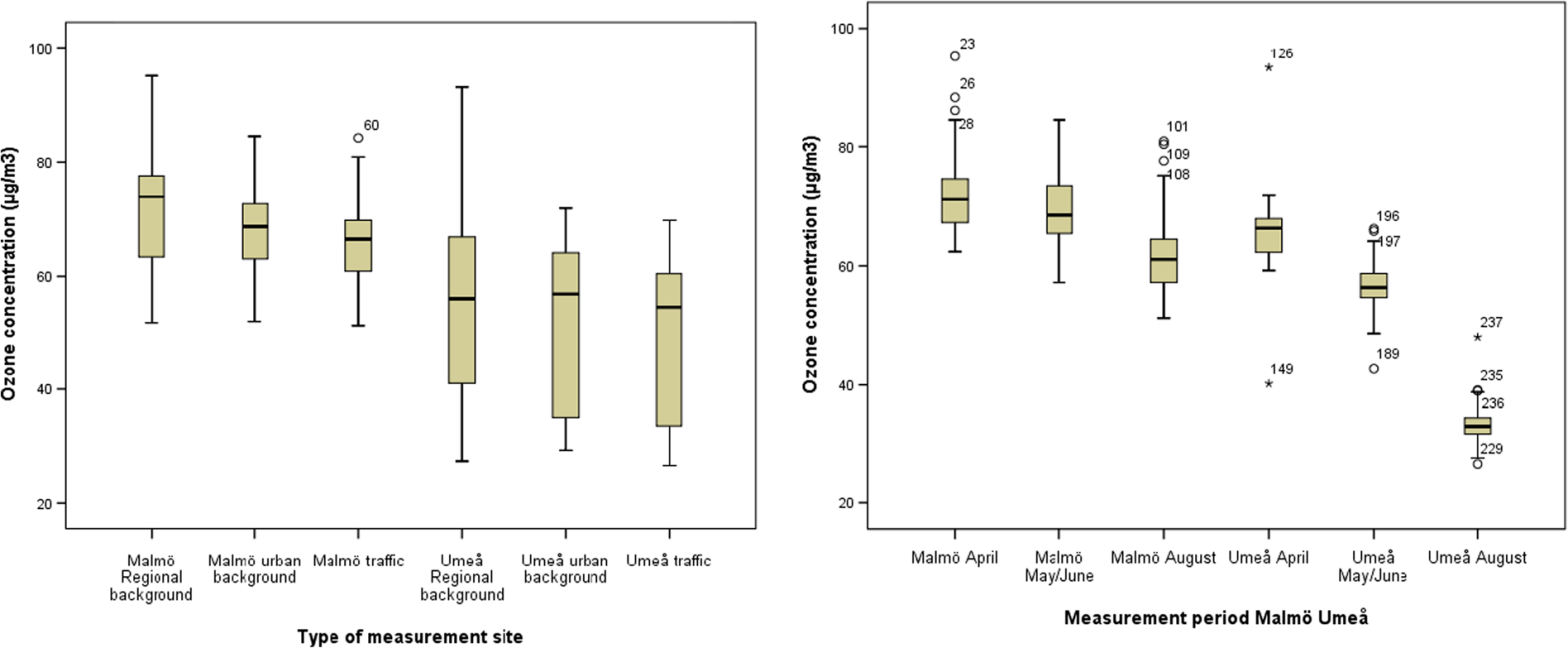
*Left:* distribution of ozone concentrations at regional background sites, urban background sites and traffic sites in the Malmö area and in the Umeå area. Median, 25th and 75th percentiles are shown in the *box*; *whiskers* indicate the 10th and 90th percentiles and individual outliers are shown as *numbered points*. *Right:* distribution of ozone concentrations for all sites in the sampling campaigns in April, May/June and August, respectively, in the Malmö area and in the Umeå area. Median, 25th and 75th percentiles are shown in the *box*; whiskers indicate the 10th and 90th percentiles and individual outliers are shown as *numbered points*

**Fig. 3:**
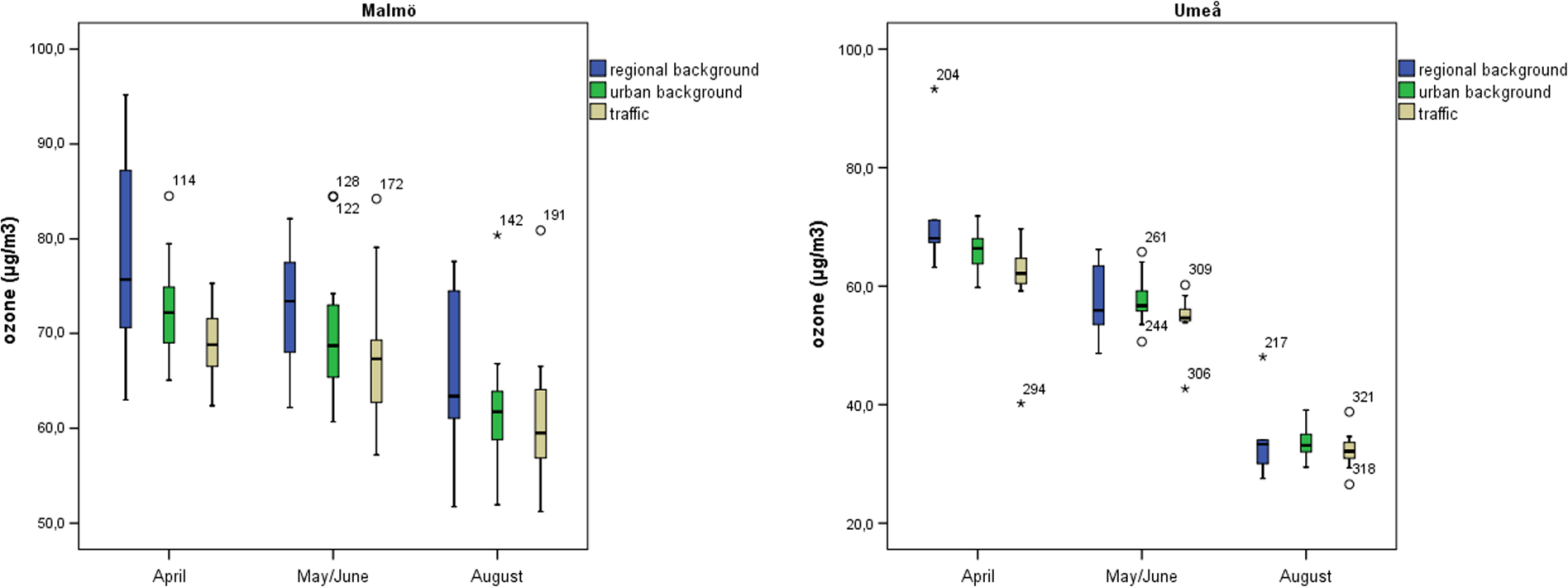
Distribution of ozone concentrations in April, May/June and August at regional background sites, at urban background sites and at traffic sites in Malmö (*left figure*) and in Umeå (*right figure*). Median, 25th and 75th percentiles are shown in the *box*, whiskers indicate the 10th and 90th percentiles and individual outliers are shown as *numbered points*

The median ozone concentration reached its maximum in April, and the lowest median concentration was found in August in both areas (Fig. 2, Supplementary Table S1 and Supplementary Table S2). Even though this phenomenon was more pronounced in the Umeå area compared to the Malmö area, the ozone concentration was statistically significantly higher in April than in August in both areas. The two areas had approximately equal range (difference between the highest and the lowest concentration) in ozone concentrations within each measurement period, with the exception of a high range of concentrations in April in Umeå (53.1 μg/m^3^) due to two deviating concentrations (Fig. 3, Supplementary Table S1 and Supplementary Table S2). However, when examining the determinants of the ozone concentration, measurement period to a much greater extent explained the variability in ozone concentration in Umeå (88%) compared to Malmö (26%) (Supplementary Table S3).

In Malmö, the ozone concentrations were statistically significantly higher at regional background sites, than at traffic sites (*p* = 0,009). The concentrations in Umeå showed the same pattern, however not statistically significant (Table 1; Figs. 2 and 3). The range in ozone concentrations within each group of measurement sites was larger in Umeå compared to Malmö, and the highest concentration range (65.8 μg/m^3^) was found at regional background sites in Umeå (Fig. 2, Supplementary Table S2). When examining the determinants of the ozone concentration, the type of measurement site influenced the variability in ozone concentration more in Malmö (9%) than in Umeå (2%) (Supplementary Table S3).

The highest ozone concentration in Malmö (95.2 μg/m^3^) was measured at a regional background site in April, and the lowest (51.2 μg/m^3^) at a heavy traffic site. There was no relation between the distance from the coast and the ozone concentrations in the Malmö region. In Umeå, the highest ozone concentration (93.3 μg/m^3^) was measured at an inland regional background site in April, which contributed to the high spatial variation in concentrations in April. One distinctive coastal site was included in the Umeå area. This site had the highest median concentration during all measurement periods (66.2 μg/m^3^) and the highest and exceptional highest concentrations in May/June and August, respectively. The lowest concentration in the Umeå area (26.5 μg/m^3^) was measured at a heavy traffic street canyon site in the city of Umeå. This site also had the lowest median concentration over all measurement periods (40.2 μg/m^3^), which contributed to the large spatial variation in ozone concentrations in Umeå.

### NO_2_, NO_*x*_ and NO

The levels of NO_2_ and NO_*x*_ were statistically significantly higher in the Malmö area, 8.1 and 12 μg/m^3^ as median concentration of all measurements (*n* = 60), compared to the Umeå area (4.5 and 8.9 μg/m^3^) (*p* < 0.001) (Table 1, Supplementary Table S1, Supplementary Table S2). The highest NO_2_ and NO_*x*_ concentrations, 40 and 103 μg/m^3^, respectively, were, however, measured in Umeå and were two to three times higher than the highest concentration in Malmö (Table 1). The concentrations were statistically significantly higher at traffic sites than at regional background sites in both cities (NO_2_: *p* = 0.000; 0.000; NO_*x*_: *p* = 0.000; *p* = 0.000 in Malmö and Umeå, respectively), however, the concentrations were similar over the different measurement periods (Table 1, Supplementary Table S1, Supplementary Table S2).

**Fig. 4:**
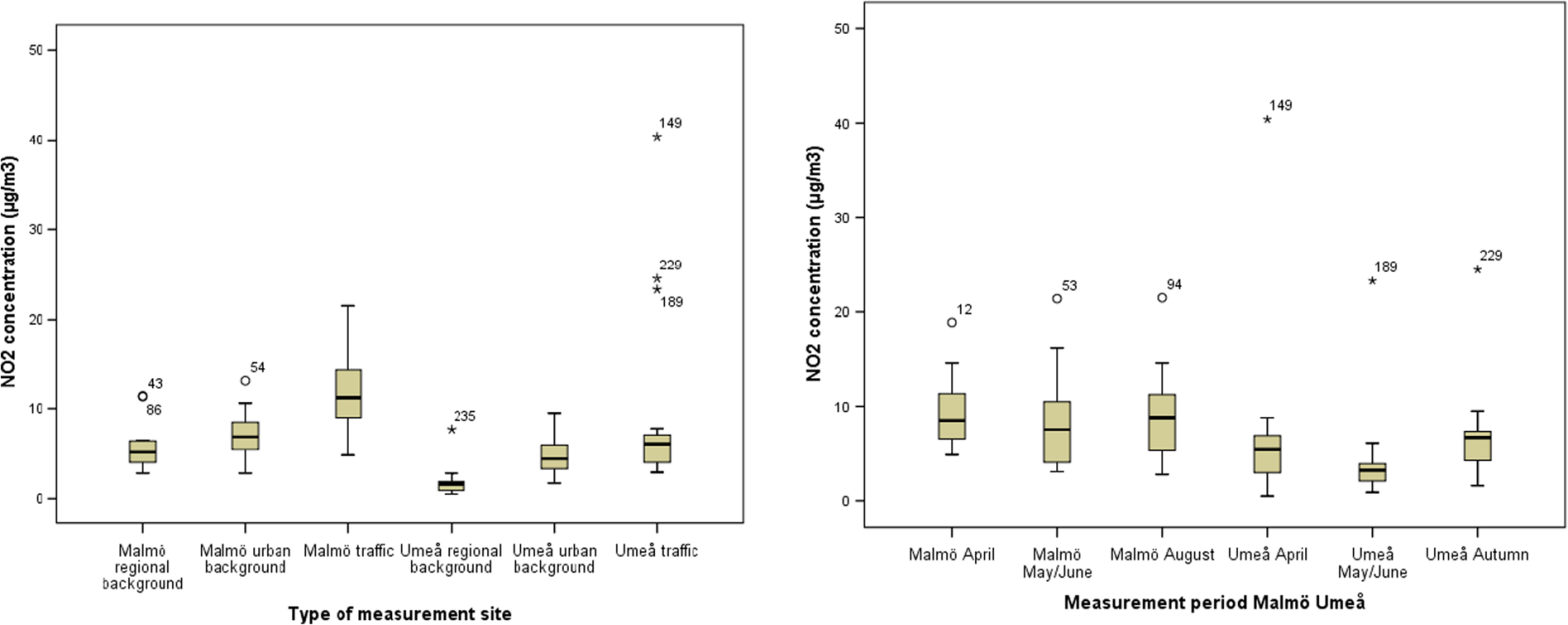
*Left:* distribution of NO_2_ concentrations at regional background sites, urban background sites and traffic sites in the Malmö area and in the Umeå area. *Right:* distribution of NO_2_ concentrations for all sites in the sampling campaigns in April, May/June and August, respectively, in the Malmö area and in the Umeå area. Median, 25th and 75th percentiles are shown in the *box*, *whiskers* indicate the 10th and 90th percentiles and individual outliers are shown as *numbered points*

**Fig. 5:**
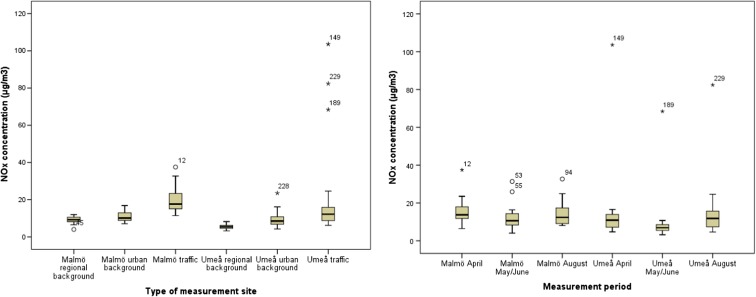
*Left*: distribution of NO_*x*_ concentrations at regional background sites, urban background sites and traffic sites in the Malmö area and in the Umeå area. *Right*: distribution of NO_*x*_ concentrations for all sites in the sampling campaigns in April, May/June and August, respectively, in the Malmö area and in the Umeå area. Median, 25th and 75th percentiles are shown in the *box*, *whiskers* indicate the 10th and 90th percentiles and individual outliers are shown as *numbered points*

The range of NO_2_ and NO_*x*_ concentrations within different groups of measurement sites was small within regional background sites and urban background sites. Within traffic sites, the range was higher, especially in Umeå (37.5 and 97.4 μg/m^3^ for NO_2_ and NO_*x*_, respectively) (Figs. 4 and 5, Supplementary Table S1 and Supplementary Table S2). The type of measurement site explained 42 and 48% of the variability in NO_2_ concentrations in Malmö and Umeå, and the corresponding figures for NO_*x*_ were 57% in Malmö and 34% in Umeå (Supplementary Table S4). When studying the range in concentrations for all measurements by measurement period, the range was generally higher in Umeå than in Malmö (Figs. 4 and 5, Supplementary Table S1 and Supplementary Table S2). Measurement period was of less importance for the variability of NO_2_ (4% in Malmö and 10% in Umeå) and NO_*x*_ (7% in Malmö and 9% in Umeå) concentrations than measurement site (Supplementary Table S4).

The median NO concentration was similar in Umeå (4.9 μg/m^3^) and in Malmö (5.0 μg/m^3^) for all measurements (Table 1, Supplementary Table S1, Supplementary Table S2). The highest NO concentration (63 μg/m^3^), though, was measured at a street canyon traffic site in Umeå and was more than three times higher than the highest NO concentration measured in Malmö (19 μg/m^3^) (Table 1). For both areas, the NO concentrations were statistically significantly higher at traffic sites and lower at regional background sites, (*p* = 0.003; 0,000; in Malmö and Umeå, respectively); however, measurement period did not influence the concentrations. The range of NO concentrations within the different groups of measurement sites was almost equal in both areas, except for the group consisting of traffic sites in Umeå for which the range was higher (61.4) (Table 1). It should be noted that as the NO concentration is derived from an indirect measurement (NO=NO_*x*_-NO_2_), the variation in NO levels is dependent of the NO_2_ and NO_*x*_ concentrations.

### Relationship between measured pollutants

The NO_2_/NO_*x*_ ratio was slightly higher in Malmö, with a median ratio of 0.6 in all groups of measurement sites and measurement periods (Supplementary Table S1). In Umeå, the median ratio was 0.3 for regional background sites, 0.5 for urban background sites and 0.4 for traffic sites (Supplementary Table S2).

The median ratio O_3_/NO_2_ for all measurements was slightly higher in Umeå (12.0) compared to Malmö (8.7), and in both cities, the ratio was several times higher at regional background sites compared to traffic sites (Supplementary Table S1, Supplementary Table S2). The median ratio O_3_/NO_2_ was highest (41.6) at the regional background sites in Umeå and almost five times higher than the median ratio at the traffic sites in this city (8.9) (Supplementary Table S2). In Malmö, the ratio was similar independent of measurement period (Supplementary Table S1). However, in Umeå, the ratio was lowest in August (5.0) and highest in May/June (17.4) (Supplementary Table S2).

## Discussion

### Ozone

The median ozone concentration was higher in the Malmö area than in the Umeå area for all measurements. During all three measurement periods, the prevailing wind in Malmö came from south-east to south-west which implicates higher ozone levels in the south of Sweden due to transport of ozone and ozone precursors from other highly polluted regions in Europe. Despite predominant winds from south and south-east, with greater distance to other polluted areas, Umeå is expected to have less long-range transport of ozone and ozone precursors. Another fact that could contribute to lower levels of ozone in Umeå might be the vicinity to forests dominated by conifer trees and birches (*Betula* sp*.)* which show high emissions of terpenes (Oderbolz et al. 2013). Studies have shown that the ozone levels are reduced over Northern Scandinavia due to ozonolysis of terpenes (oxidation of terpenes by ozone) (Curci et al. 2009).

Continuous measurements of ozone concentrations at a number of regional background sites in Sweden support this, showing a significantly higher number of episodes of high concentrations in the southern part of Sweden than in the north due to transport of ozone from other parts of Europe. The Swedish air quality limit as an 8-h mean for ozone is 120 μg/m^3^, set to protect human health. There is an indication of a downward trend of exceedances at regional background sites in Sweden, but the variation in ozone levels from 1 year to another is large, so the trend is unclear. Our measured levels over three measurement periods in 2012, 67 and 56 μg/m^3^ in Malmö and Umeå, respectively, are comparable to the annual mean measured by continuous ozone monitors, the same year, 60 and 56 μg/m^3^, near Malmö and Umeå (Sjöberg et al. 2014).

The Swedish environmental quality objective “Clean Air” has specified a target value, taking sensitive groups into consideration, which states that the ground-level ozone should not exceed 70 μg/m^3^ calculated as an 8-h mean and 80 μg/m^3^ as an hourly mean. These target values are greatly exceeded in all regions in Sweden, even in the northern part (Sjöberg et al. 2014). Our measurements over 1 week showed the highest concentration of 95 μg/m^3^, which indicates hourly levels exceeding the target value.

The ozone concentration was highest in April and lowest in August in both areas, but this pattern was more pronounced in Umeå than in Malmö. This is in accordance with other studies that found an ozone maximum in spring (Clapp and Jenkin 2001; Scheel et al. 1997). Scheel et al. found that the annual ozone maximum was shifted from spring in the north of Europe (79° N) to late summer in Austria and Hungary (Scheel et al. 1997). The ozone concentration has an annual variation, and hypothetically, the highest concentration should be found at the solar maximum, which occurs in summer in the Northern Hemisphere. However, historical time series ozone measurements taken at Arkona on the Baltic coast, at Montsouris in Paris, France, and in Athens showed a clear spring maximum peak (Monks 2000). In a German study, Scheel et al. showed that the annual ground-level maximum in northern Europe occurred in spring, while in south-eastern Europe, the maximum concentrations were found in late summer (Scheel et al. 1997). The spring ozone maximum is a phenomenon characteristic of the Northern Hemisphere (Scheel et al. 1997) and is explained by several factors, of which one of the causes may be the stratospheric–tropospheric ozone exchange in spring (Monks 2000). However, the main reason is suggested to be the accumulation of peroxyacetyl nitrate (PAN), carbon dioxide and non-methane volatile organic compounds (VOCs) in combination with increased solar radiation, temperature and emissions of biogenic isoprene, which altogether leads to a photochemically driven spring maximum (Gibson et al. 2009). The continuous measurements of ozone levels in Sweden also show the highest concentrations in spring (March–May) with a maximum arriving slightly earlier in the northern part than in the southern part of the country. These measurements show that during the year our study took part, 2012, the maximum ozone concentration near Umeå (Vindeln) was found in March, while near Malmö (Vavihill), the ozone maximum occurred 2 months later (Sjöberg et al. 2014).The aforementioned German study also showed, from ozone measurement data collected from 25 European sites during 1989 to 1993, a summertime ozone gradient with increasing ozone levels from the north-west of Europe to the south-east.

The ozone concentrations were highest at the regional background sites, decreasing at urban background sites and lowest at traffic sites in both cities. Other studies have found the same pattern, with low concentrations at traffic sites and higher concentrations at rural sites (Im et al. 2013; Melkonyan and Kuttler 2012; Syri et al. 2001). This is explained by the rapid reaction between NO and O_3_ close to the points of emission. At traffic sites, the dominant NO_*x*_ emission from vehicle exhaust is NO. A consequence of the reaction between NO and O_3_ is that O_3_ is consumed and ozone levels here are lower.

#### Determinants of variability in O_3_ levels

Although we measured ozone during April, May/June and late August, the measurement period explained the major part (38%) of the variability in the ozone concentration for both cities, whereas the city (Malmö or Umeå) explained 32% of the variability (Supplementary Table S3). City and measurement period together explained 70% of the variability, whereas type of measurement site only explained 2% of the variability in the ozone concentration (Supplementary Table S3). In Umeå, type of measurement site and measurement period together explained 89% of the variability in ozone concentrations, whereas the same variables explained only 35% in Malmö (Supplementary Table S3). One possible reason may be that the ozone spring maximum is more pronounced in the north of Sweden than in the south. Another reason may be transport of ozone and ozone precursors from other parts of Europe, affecting the levels of ozone at all measurement periods and all types of measurement sites more in southern Sweden than in the north.

### NO_2_, NO_*x*_ and NO

The mean concentrations of NO_2_, measured over three measurement periods, were low (8.8 and 5.7 μg/m^3^ in Malmö and Umeå, respectively) in view of the Swedish air quality limit of 40 μg/m^3^ as an annual mean. The concentrations were highest at traffic sites and lowest at regional sites, in both regions. This is expected, as the most important anthropogenic sources of NO_*x*_ are on-road vehicles, working machines and energy production. With an increased distance from the main sources, the concentrations at regional background sites are low in Sweden. As part of Sweden’s environmental monitoring programme, NO_2_ concentrations are measured continuously at a number of regional background sites around the country. The concentrations have decreased over the last decade, and the levels show a clear south–north gradient in which the concentration of NO_2_ at several measurement sites in the north of Sweden represents about 15% of the levels in the south. This is due to transportation of NO_2_ from Central Europe to the southern part of Sweden (Naturvårdsverket 2016). The mean annual concentration of NO_2_ at a regional background site in the vicinity of Malmö in 2012 (Vavihill) was 4.9 μg/m^3^. In northern Sweden, the corresponding annual mean 2012 in Rickleå was 1.5 μg/m^3^ (Naturvårdsverket 2016). In our study, the mean concentration of NO_2_ at regional background sites showed the same pattern, with a mean concentration of 5.9 μg/m^3^ in the Malmö area and 2.0 μg/m^3^ in the Umeå area. In 2012, Malmö had twice as many passenger cars and lorries as Umeå (Transport Analysis 2016), which may explain the higher NO_2_ and NO_*x*_ concentrations in Malmö. Interesting to notice, however, is that the highest NO_2_ concentration (40 μg/m^3^) was measured at a street canyon traffic site in Umeå, the smaller of the two cities and situated in the north of Sweden (Table 1). This was twice as high as the highest concentration measured in Malmö.

#### Determinants of variability in NO_2_ and NO_*x*_ levels

Unlike the case with ozone, the variable explaining the major part (31%) of the variability in the NO_2_ concentrations for both cities was type of measurement site, whereas measurement period explained 5% of the variability (Supplementary Table S4). For NO_*x*_, the type of measurement site was an even more important factor explaining 36% of the variability in concentrations for both cities, while measurement period explained 7% of the variability. The city (Malmö or Umeå) was explaining more of the variability in NO_2_ (19%) than in NO_*x*_ concentrations (5%)_._ Together, city and type of measurement site explained 52 and 42% of the variability in NO_2_ and NO_*x*_ concentrations, respectively (Supplementary Table S4).

This study was carried out in April, May/June and early August, which explains the minor impact of measurement period on NO_2_ and NO_*x*_ concentrations. If the measurements also had included the winter season, the concentrations probably would have been higher due to very low temperature, inversion, increased emissions from cold-started vehicles and residential heating, and measurement period would have been more important to explain the concentrations. The impact of these factors is more pronounced in the northern part of Sweden, why including also the winter months would have increased the concentrations of NO_2_ and NO_*x*_ more in Umeå than in Malmö.

Together, measurement period and type of measurement site explained 45 and 59% of the variability in NO_2_ concentrations in Malmö and Umeå, respectively, whereas the corresponding figures for the variability in NO_*x*_ concentrations were 63 and 44% in Malmö and Umeå, respectively (Supplementary Table S4). This might be explained by the odd fact that Umeå had higher NO concentrations at some sites, while both NO_*x*_ and NO_2_ concentrations were higher in Malmö.

### Relationship between measured pollutants

The median NO_2_/NO_*x*_ ratio was slightly higher in Malmö than in Umeå and the same for all site types. The vehicle fleet size, twice as many in Malmö as in Umeå, together with the higher levels of ozone to react with NO to form NO_2_, might explain the higher ratio in Malmö.

In Umeå, the NO_2_/NO_*x*_ ratio was slightly smaller at traffic sites than at urban background sites in accordance with previously published results from the ESCAPE study (Cyrys et al. 2012). The higher ratio at urban background sites indicates reactions of primary emitted NO to NO_2_ to increase at greater distance to busy streets.

The moderate difference in ratios between traffic sites and urban background sites might reflect the composition of the vehicle fleet. It is known that modern diesel cars emit higher amounts of primary NO_2_ compared to petrol-fuelled cars, which increase the NO_2_/NO_*x*_ ratio (Grice et al. 2009). The proportion of diesel cars in Sweden more than doubled during the period 2008 to 2012 (Transport Analysis 2016) and should have a significant effect upon the NO_2_ levels, especially at road-side locations. Besides, the use of particulate filters to reduce particle emissions in diesel vehicles increases the NO_2_/NO_*x*_ ratio, as some of the particulate filters are based on oxidation of NO to NO_2_ (Grice et al. 2009; Wild et al. 2017).

As expected, the ratio O_3_/NO_2_ was highest in regional background and most pronounced in Umeå (Supplementary Table S2).

### Strengths and weaknesses

As mentioned in the “Introduction”, to our knowledge, there is no study that has measured ozone and NO_*x*_ simultaneously at so many sites (*n* = 20), with repeated measurements in three measurement periods in two parts of a country. This enabled the study of ratios between ozone and NO_2_, which are important for the understanding of ozone levels in different places. It would have been interesting also to include winter measurements, assuming higher NO_*x*_ levels and very low ozone levels. As the climate in the two study areas is different, there might be some seasonal lag between the two study areas. This could be an issue when comparing measurement periods between the two areas. Ogawa samplers used in the study have been used in earlier studies (Gibson et al. 2009; Hauser et al. 2015; Jerrett et al. 2009; Mukerjee et al. 2009) and are considered to be a reliable technique to measure ozone and NO_*x*_ (Bhangar et al. 2013; Hagenbjork-Gustafsson et al. 2010; Sather et al. 2007).

## Conclusion

In both cities, the highest ozone levels were found in April, and Malmö had higher levels of ozone in comparison to Umeå. We found a considerable spatial variation in ozone concentrations within the two city areas. The ozone levels as well as the range of measured concentrations were highest at regional background sites in both cities. For NO_2_ and NO_*x*,_ Malmö showed higher median levels than Umeå. However, there were no difference in concentrations between the different measurement periods in either of the cities. The spatial variation in NO_2_ concentrations within each area was smaller than the variation in ozone concentrations in both cities. For ozone, the measurement period had a greater impact on variability in concentrations than type of measurement site. For NO_2_ and NO_*x*,_ the type of measurement site explained most of the variability in concentrations, while measurement period was of less importance. The importance of measurement period as an explanatory variable for NO_2_ and NO_*x*_ concentrations would have increased if the measurements had also included the winter months. The median NO_2_/NO_*x*_ ratio was 0.6 in Malmö and 0.4 in Umeå.

## Electronic supplementary material

ESM 1 (DOCX 21 kb).

## References

[CR1] Bhangar S, Singer BC, Nazaroff WW (2013). Calibration of the Ogawa passive ozone sampler for aircraft cabins. Atmospheric Environment.

[CR2] Carslaw DC (2005). Evidence of an increasing NO_2_/NO_*x*_ emissions ratio from road traffic emissions. Atmospheric Environment.

[CR3] Clapp LJ, Jenkin ME (2001). Analysis of the relationship between ambient levels of O_3_, NO_2_ and NO as a function of NO_*x*_ in the UK. Atmospheric Environment.

[CR4] Coyle M, Smith RI, Stedman JR, Weston KJ, Fowler D (2002). Quantifying the spatial distribution of surface ozone concentration in the UK. Atmospheric Environment.

[CR5] Curci G, Beekmann M, Vautard R, Smiatek G, Steinbrecher R, Theloke J, Friedrich R (2009). Modelling study of the impact of isoprene and terpene biogenic emissions on European ozone levels. Atmospheric Environment.

[CR6] Cyrys J, Eeftens M, Heinrich J, Ampe C, Armengaud A, Beelen R (2012). Variation of NO_2_ and NO_*x*_ concentrations between and within 36 European study areas: results from the ESCAPE study. Atmospheric Environment.

[CR7] Entwistle J, Weston K, Singles R, Burgess R (1997). The magnitude and extent of elevated ozone concentrations around the coasts of the British Isles. Atmospheric Environment.

[CR8] Garcia MA, Sanchez ML, Perez IA, de Torre B (2005). Ground level ozone concentrations at a rural location in northern Spain. Science of the Total Environment.

[CR9] Gibson MD, Guernsey JR, Beauchamp S, Waugh D, Heal MR, Brook JR (2009). Quantifying the spatial and temporal variation of ground-level ozone in the rural Annapolis Valley, Nova Scotia, Canada using nitrite-impregnated passive samplers. Journal of the Air & Waste Management Association.

[CR10] Grice S, Stedman J, Kent A, Hobson M, Norris J, Abbott J (2009). Recent trends and projections of primary NO_2_ emissions in Europe. Atmospheric Environment.

[CR11] Hagenbjork-Gustafsson A, Tornevi A, Forsberg B, Eriksson K (2010). Field validation of the Ogawa diffusive sampler for NO_2_ and NO_*x*_ in a cold climate. Journal of Environmental Monitoring.

[CR12] Hauser CD, Buckley A, Porter J (2015). Passive samplers and community science in regional air quality measurement, education and communication. Environmental Pollution.

[CR13] Im U, Incecik S, Guler M, Tek A, Topcu S, Unal YS (2013). Analysis of surface ozone and nitrogen oxides at urban, semi-rural and rural sites in Istanbul, Turkey. Science of the Total Environment.

[CR14] Jerrett M, Finkelstein MM, Brook JR, Arain MA, Kanaroglou P, Stieb DM (2009). A cohort study of traffic-related air pollution and mortality in Toronto, Ontario, Canada. Environmental Health Perspectives.

[CR15] Klingberg J, Karlsson PE, Karlsson GP, Hu Y, Chen D, Pleijel H (2012). Variation in ozone exposure in the landscape of southern Sweden with consideration of topography and coastal climate. Atmospheric Environment.

[CR16] Malmqvist E, Olsson D, Hagenbjork-Gustafsson A, Forsberg B, Mattisson K, Stroh E (2014). Assessing ozone exposure for epidemiological studies in Malmo and Umea, Sweden. Atmospheric Environment.

[CR17] Mazzeo NA, Venegas LE, Choren H (2005). Analysis of NO, NO_2_, O_3_ and NO_*x*_ concentrations measured at a green area of Buenos Aires City during wintertime. Atmospheric Environment.

[CR18] Melkonyan A, Kuttler W (2012). Long-term analysis of NO, NO_2_ and O_3_ concentrations in North Rhine-Westphalia, Germany. Atmospheric Environment.

[CR19] Monks PS (2000). A review of the observations and origins of the spring ozone maximum. Atmospheric Environment.

[CR20] Mukerjee S, Oliver KD, Seila RL, Jacumin HH, Croghan C, Daughtrey EH (2009). Field comparison of passive air samplers with reference monitors for ambient volatile organic compounds and nitrogen dioxide under week-long integrals. Journal of Environmental Monitoring.

[CR21] Naturvårdsverket. (2016). Swedish Environmental Protection Agency. (http://www.naturvardsverket.se/Sa-mar-miljon/Statistik-A-O/Kvavedioxid-halter-i-luft-regional-bakgrund). Retrieved 20 January 2016.

[CR22] Notario A, Bravo I, Adame JA, Diaz-de-Mera Y, Aranda A, Rodriguez A (2013). Variability of oxidants (OX = O_3_+ NO_2_), and preliminary study on ambient levels of ultrafine particles and VOCs, in an important ecological area in Spain. Atmospheric Research.

[CR23] Oderbolz DC, Aksoyoglu S, Keller J, Barmpadimos I, Steinbrecher R, Skjøth CA (2013). A comprehensive emission inventory of biogenic volatile organic compounds in Europe: improved seasonality and land-cover. Atmospheric Chemistry and Physics.

[CR24] Piikki K, Klingberg J, Karlsson GP, Karlsson PE, Pleijel H (2009). Estimates of AOT ozone indices from time-integrated ozone data and hourly air temperature measurements in southwest Sweden. Environmental Pollution.

[CR25] R Core Team. (2017). R: A language and environment for statistical computing. R Foundation for Statistical Computing, Vienna, Austria. URL https://www.R-project.org/.

[CR26] Sather ME, Slonecker ET, Mathew J, Daughtrey H, Williams DD (2007). Evaluation of Ogawa passive sampling devices as an alternative measurement method for the nitrogen dioxide annual standard in El Paso, Texas. Environmental Monitoring and Assessment.

[CR27] Scheel HE, Areskoug H, Geiss H, Gomiscek B, Granby K, Haszpra L (1997). On the spatial distribution and seasonal variation of lower-troposphere ozone over Europe. Journal of Atmospheric Chemistry.

[CR28] Sjöberg K., B. L. E. Karlsson, Pihl G., Danielsson H. (2014). *Nr C 53, Sakrapport 2013 Data från övervakning inom Programområde Luft t.o.m. år 2013*.

[CR29] SMHI. (2016). Swedish Meteorological and Hydrological Institute. (http://www.smhi.se) Open data. Retrieved 8 February, 2016.

[CR30] Song F, Shin JY, Jusino-Atresino R, Gao Y (2011). Relationships among the springtime ground-level NO_*x*_, O_3_ and NO_3_ in the vicinity of highways in the US East Coast. Atmospheric Pollution Research.

[CR31] Sundberg J, Karlsson PE, Schenk L, Pleijel H (2006). Variation in ozone concentration in relation to local climate in south-west Sweden. Water Air and Soil Pollution.

[CR32] Syri S, Amann M, Schopp W, Heyes C (2001). Estimating long-term population exposure to ozone in urban areas of Europe. Environmental Pollution.

[CR33] Transport Analysis (2016). A Swedish government agency for transport policy analysis. (www.trafa.se/vagtrafik/fordon) Retrieved 10 February, 2016.

[CR34] Vingarzan R (2004). A review of surface ozone background levels and trends. Atmospheric Environment.

[CR35] WHO (2008). *Health risks of ozone from long-range transboundary air pollution*.

[CR36] Wild RJ, Dubé WP, Aikin KC, Eilerman SJ, Neuman JA, Peischl J (2017). On-road measurements of vehicle NO2/NO_*x*_ emission ratios in Denver, Colorado, USA. Atmospheric Environment.

