# Supplementary material for: The spatial variation of O3, NO, NO2 and NOx and the relation between them in two Swedish cities
Source: Environ Monit Assess. 2017 Mar 13;189(4):161. doi: 10.1007/s10661-017-5872-z (PMC5348563; doi:10.1007/s10661-017-5872-z)
Supplement: Supplementary file 1 — (DOCX 21 kb). [file 10661_2017_5872_MOESM1_ESM.docx]

**Contents of Supplementary information**

Table S 1. Arithmetic mean (AM), (standard deviation), median (min-max), and geometric mean (GM) of measured pollutants, O_3_/NO_2_ and NO_2_/NO_x_ ratios in different environments for all measurements in Malmö.

Table S 2. Arithmetic mean (AM), (standard deviation), median (min-max), and geometric mean (GM) of measured pollutants, O_3_/NO_2_ and NO_2_/NO_x_ ratios in different environments for all measurements in Umeå.

Table S 3. Determinants of measured ozone concentrations in both cities (Malmö and Umeå), and in Malmö and Umeå.

Table S 4. Determinants of measured NO_2_ and NO_x_ concentrations in both cities (Malmö and Umeå), and in Malmö and Umeå.

|  | **Malmö** | | | | | | | |
| --- | --- | --- | --- | --- | --- | --- | --- | --- |
|  |  | Regional background sites  (n=21) | Urban background sites  (n=42) | Traffic sites  (n=57) | Measurement period 1, April  (n=40) | Measurement period 2, May/June  (n=40) | Measurement period 3, August  (n=40) | All measurements  (n=120) |
| O_3_  (µg/m^3^) | AM (s.d) | 72.5 (10.5) | 68.5 (7.7) | 65.8(6.8) | 72.0 (7.0) | 69.6 (7.1) | 62.2 (7.1) | 67.9 (8.1) |
|  | Median (min-max) | 73.9, (51.7-95.2) | 68.6, (51.9-84.5) | 66.4 (51.2-84.2) | 71.2 (62.4-95.2) | 68.5 (57.2-84.5) | 61.0 (51.2-80.9) | 67.1 (51.2 - 95.2) |
|  | GM | *71.7* | *68.0* | *65.5* | *71.7* | *69.2* | *61.8* | 67.4 |
|  |  | Regional background sites (n=12) | Urban background sites  (n=21) | Traffic sites  (n=27) | Measurement period 1, April  (n=20) | Measurement period 2, May/June  (n=20) | Measurement period 3, August  (n=20) | All measurements  (n=60) |
| NO_2_  (µg/m^3^) | Mean concentration (s.d) | 5.9 (2.8) | 6.8 (2.5) | 11.9 (4.3) | 9.4 (3.6) | 8.1 (5.0) | 8.9 (4.6) | 8.8 (4.3) |
|  | Median (min-max) | 5.2(2.8-11.4) | 6.6 (2.8-13.2) | 11.2 (4.8-21.5) | 8.5 (4.9-18.9) | 7.2 (3.1-21.4) | 8.4 (2.8-21.5) | 8.1 (2.8 - 21.5) |
|  | GM | 5.3 | 6.4 | 11.2 | 8.8 | 6.9 | 7.8 | 7.8 |
| NO_x_  (µg/m^3^) | AM (s.d) | 9.0 (2.2) | 10.6 (2.6) | 19.3 (6.8) | 15.4 (6.9) | 12.2 (6.6) | 14.3 (6.6) | 14.0 (6.7) |
|  | Median (min-max) | 9.2 (4.0-12.0) | 9.5 (7.1-16.7) | 17.6 (11.4-37.5) | 13.8 (6.5-37.5) | 10.5 (4.0-31.4) | 11.5 (8.1-32.7) | 12.0  (4.0 – 37.5) |
|  | GM | 8.7 | 10.3 | 18.3 | 14.3 | 11.0 | 13.1 | 12.7 |
| NO  (µg/m^3^) | AM (s.d) | 3.3 (2.1) | 4.0 (2.5) | 7.4 (4.3) | 6.0 (3.8) | 4.5 (4.1) | 5.5 (3.4) | 5.3 (3.9) |
|  | Median (min-max) | 3.9 (0.6-5.6) | 4.2 (0.6-11.2) | 7.3 (0.6-18.6) | 5.3 (1.0-18.6) | 4.2 (0.6-16.8) | 5.3 (0.6-12.1) | 5.0  (0.6-18.6) |
|  | GM | 2.4 | 3.0 | 5.9 | 5.1 | 2.7 | 4.0 | 3.8 |
| Ratio O_3_/NO_2_ | AM (s.d) | 13.5 (5.7) | 11.8 (6.5) | 6.4 (2.7) | 8.5 (3.0) | 11.8 (6.5) | 9.2 (5.5) | 9.8 (5.3) |
|  | Median (min-max) | 12.1 (4.6-23.7) | 10.7 (3.2-23.7) | 5.8 (2.8-14.4) | 8.1 (3.4-15.4) | 10.7 (3.2-23.7) | 8.0 | 8.7 (2.8-23.7) |
|  | GM | 12.3 | 10.0 | 6.0 | 8.0 | 10.0 | 7.9 | 8.6 |
| Ratio NO_2_/ NO_x_ | AM (s.d) | 0.7 (0.3) | 0.7 (0.2) | 0.6 (0.2) | 0.6 (0.1) | 0.7 (0.3) | 0.6 (0.2) | 0.6 (0.2) |
|  | Median (min-max) | 0.6 (0.3-1.1) | 0.6 (0.3-1.3) | 0.6 (0.4-1.0) | 0.6 (0.5-0.9) | 0.6 (0.4-1.3) | 0.6 (0.3-1.1) | 0.6 (0.3-1.3) |
|  | GM | 0.6 | 0.6 | 0.6 | 0.6 | 0.6 | 0.6 | 0.6 |

Table S 1. Arithmetic mean (AM), (standard deviation), median (min-max), and geometric mean (GM) of O_3_, NO_2_, NO_x_, NO, O_3_/NO_2_ and NO_2_/NO_x_ ratios in different environments for all measurements in Malmö.

|  | **Umeå** | | | | | | | |
| --- | --- | --- | --- | --- | --- | --- | --- | --- |
|  |  | Regional background sites (n=27) | Urban background sites  (n=57) | Traffic sites  (n=36) | Measurement period 1, April  (n=40) | Measurement period 2, May/June  (n=40) | Measurement period 3, August  (n=40) | All measurements  (n=120) |
| O_3_  (µg/m^3^) | AM (s.d) | 54.3 (16.4) | 52.3 (14.3) | 49.3 (13.5) | 65.6 (7.0) | 56.6 (4.3) | 33.4 (3.7) | 51.8 (14.6) |
|  | Median (min-max) | 57.2 (27.5-93.3) | 56.6 (29.4-71.8) | 54.4 (26.5-69.7) | 66.3 (40.2-93.3) | 56.4 (42.7-66.2) | 32.8 (26.5-48) | 56.1 (26.5 – 93.3) |
|  | GM | 51.7 | 50.1 | 47.3 | 65.2 | 56.4 | 33.2 | 49.6 |
|  |  | Regional background sites  (n=13) | Urban background sites  (n=17) | Traffic sites  (n=30) | Measurement period 1, April  (n=20) | Measurement period 2, May/June  (n=20) | Measurement period 3, August  (n=20) | All measurements    (n=60) |
| NO_2_  (µg/m^3^) | Mean concentration (s.d) | 2.0 (1.8) | 4.8 (2.1) | 7.9 (7.9) | 6.7 (8.3) | 4.1 (4.7) | 6.5 (4.8) | 5.7 (6.2) |
|  | Median (min-max) | 1.6 (0.5-7.7) | 4.4 (1.7-9.5) | 6.1 (2.9-40.4) | 5.5 (0.5-40.4) | 3.3 (0.9-23.3) | 6.7 (1.6-24.5) | 4.5 (0.5 – 40.4) |
|  | GM | 1,5 | 4.4 | 6.2 | 4.5 | 3.0 | 5.4 |  |
| NO_x_  (µg/m^3^) | AM (s.d) | 5.4 (1.2) | 9.7 (4.6) | 19.3 (23.0) | 15.2 (21.1) | 10.0 (13.9) | 15.3 (16.8) | 13.5 (17.4) |
|  | Median (min-max) | 5.4 (3.2-8.2) | 8.5 (4.2-23.5) | 12.2 (6.2-103.6) | 11.0 (4.7-103.6) | 7.0 (3.2-68.4) | 11.9 (4.6-82.4) | 8.9  (3.2 – 103.6) |
|  | GM | 5.3 | 8.9 | 13.9 | 11.2 | 7.5 | 11.7 | 9.9 |
| NO  (µg/m^3^) | AM (s.d) | 3.7 (1.3) | 4.9 (2.9) | 11.3 (15.4) | 8.6 (13.0) | 6.0 (9.3) | 9.0 (12.2) | 7.9 (11.5) |
|  | Median (min-max) | 3.8 (0.6-5.4) | 4.3 (0.6-14.0) | 6.3 (1.8-63.2) | 5.6 (3.1-63.2) | 4.0 (0.6-45.1) | 5.8 (0.6-57.9) | 4.9 (0.6 – 63.2) |
|  | GM | 3.3 | 4.2 | 7.3 | 6.2 | 4.1 | 5.7 | 5.3 |
| Ratio O_3_/NO_2_ | AM (s.d) | 45.0 (33.5) | 12.7 (7.7) | 9.5 (5.5) | 22.2 (29.4) | 24.6 (19.1) | 7.5 (5.6) | 18.1 (21.6) |
|  | Median (min-max) | 41.6 (5.1-134.8) | 10.3 (3.9-33.9) | 8.9 (1.0-20.8) | 11.9 (1.0-134.8) | 17.4 (1.8-66.0) | 5.0, (1.1-21.2) | 12.0 (1.0-134.8) |
|  | GM | 34.4 | 10.8 | 7.6 | 13.9 | 18.7 | 6.0 | 11.6 |
| Ratio NO_2_/ NO_x_ | AM (s.d) | 0.4 (0.3) | 0.5 (0.2) | 0.5 (0.1) | 0.4 (0.1) | 0.5 (0.3) | 0.5 (0.3) | 0.5 (0.2) |
|  | Median (min-max) | 0.3 (0.1-1.5) | 0.5 (0.4-1.1) | 0.4 (0.3-0.8) | 0.5 (0.1-0.6) | 0.4 (0.2-1.1) | 0.4 (0.3-1.5) | 0.4 (0.1-1.5) |
|  | GM | 0.3 | 0.5 | 0.4 | 0.4 | 0.4 | 0.5 | 0.4 |

Table S 2. Arithmetic mean (AM), (standard deviation), median (min-max), and geometric mean (GM) of O_3_, NO_2_, NO_x_, NO, O_3_/NO_2_ and NO_2_/NO_x_ ratios in different environments for all measurements in Umeå.

| **O_3_** | Measurement  period | Type of measurement site | City | Measurement  period AND city | Measurement  period AND Type of measurement site | Measurement  period AND City AND Type of measurement site |
| --- | --- | --- | --- | --- | --- | --- |
| Both cities | 38 % | 2 % | 32 % | 70 % |  | 72 % |
| Malmö | 26 % | 9 % |  |  | 35 % |  |
| Umeå | 88 % | 2 % |  |  | 89 % |  |

Table S 3. Determinants of measured ozone concentrations in both cities (Malmö and Umeå), and in Malmö and Umeå.

| **NO_2_** | Measurement  period | Type of measurement site | City | City AND Type of measurement site | Measurement  period AND Type of measurement site | Measurement  period AND City AND Type of measurement site |
| --- | --- | --- | --- | --- | --- | --- |
| Both cities | 5 % | 31 % | 19 % | 52 % |  | 57 % |
| Malmö | 4 % | 42 % |  |  | 45 % |  |
| Umeå | 10 % | 48 % |  |  | 59 % |  |
| **NO_x_** |  |  |  |  |  |  |
| Both cities | 7 % | 36 % | 5 % | 42 % |  | 49 % |
| Malmö | 7 % | 57 % |  |  | 63 % |  |
| Umeå | 9 % | 34 % |  |  | 44 % |  |

Table S 4. Determinants of measured NO_2_ and NO_x_ concentrations in both cities (Malmö and Umeå), and in Malmö and Umeå.
